# Announcement: Metals in Medicine Workshop

**DOI:** 10.1155/MBD.1998.116

**Published:** 1998


					METALS IN MEDICINE WORKSHOP
A workshop covering topics related to the use of Metal Complexes in Medicine will be held
as an applications day of the
IXth International Conference ofthe Chemistry and Organometallic Chemistry of
Germanium, Tin, and Lead
Location: World Congress Centre, Melbourne
Date: Wednesday September 23rd, 1998
Speakers who have agreed to participate are:
Plenary lecturer: Jan Reedijk (Leiden University)
Contributing Speakers: Sue Berners-Price (Griffith University), Trevor Hambley (The
University of Sydney), Margaret Harding (The University of Sydney), V.G. Kumar Das
(Universiti Malaysia), Edmunds Lukevics (Latvian Institute of Organic Synthesis), Michael
Millward (Peter MacCallum Cancer Institute), Valery Petrosyan (Lomonosov University),
Lou Rendina (The University of Adelaide), Youn Soo Sohn (Korea Institute of Science and
Technology), Dick de Vos (Pharmachemie BV, Haarlem) and Lorraine Webster (Peter
MacCallum Cancer Institute)
Tentative Program:
9:30 Welcome
9:35 Plenary lecture
10:30 Catered morning tea
11:00 Lectures
12:20 Catered lunch
1:30 Lectures
3:00 Catered afternoon tea
3:30 Lectures
4:10 Poster session with catered refreshments
6:00 Close
116
Proceedings" The proceedings of the Metals in Medicine workshop will be published in
Metal-Based Drugs. All participants (oral and poster presentations) are invited to contribute
to the dedicated issue(s) of Metal-Based Drugs. The proceedings will be distributed at the
Metals in Medicine workshop and hence, strict deadlines for submission of articles/abstracts
will be adhered to.
All contributions should be sent to Dr ERT Tiekink
Department of Chemistry,
The University of Adelaide,
AUSTRALIA 5005
etiekink@chemistry,adelaide.edu.au
The deadline for all contributions to be included in the Metals in Medicine proceedings is
June 30th, 1998.
Instructions for authors are attached.
All papers will be subject to the normal refereeing procedures. Submissions should be
submitted electronically wherever possible. Reprints will be distributed free of charge to the
principal author.
Participants who wish to present a poster are advised that the poster space is 1 rn wide and
1.5 rn high.
Registration"
The all inclusive (morning/afternoon teas, lunch, poster session and proceedings) cost for
the Metals in Medicine workshop will be $AUD 150 ($AUD 90 for students, unemployed,
retired participants). A registration form is attached. Other details (i.e. about Melbourne,
accommodation, etc) may be downloaded from our website or obtained from Dr Tiekink at
the above address.
Web site:
http://www.science.adelaide.edu.au/chemist/conferences/ICCOcGTL9/mmw.htm
117

				

